# Whole Transcriptome Profiling of the Effects of Cadmium on the Liver of the Xiangxi Yellow Heifer

**DOI:** 10.3389/fvets.2022.846662

**Published:** 2022-04-14

**Authors:** Yameng Wei, Kangle Yi, Caomeihui Shen, Xue Chen, Tariq Iqbal, Maosheng Cao, Tong Chen, Yang Luo, Jianbo Li, Xu Zhou, Chunjin Li, Lu Chen

**Affiliations:** ^1^College of Animal Sciences, Jilin University, Changchun, China; ^2^Grassland and Herbivore Research Laboratory, Hunan Animal Husbandry and Veterinary Research Institute, Changsha, China

**Keywords:** RNA-seq, cadmium, liver, gene ontology, Kyoto Encyclopedia of Genes

## Abstract

Cadmium (Cd) is a major heavy metal toxicant found in industrial zones. Humans and animals are exposed to it through their diet, which results in various physiological problems. In the current study, the toxic effects of Cd on the liver were investigated by whole-transcriptome sequencing (RNA-seq) of the livers of Xiangxi heifers fed a diet with excess Cd. We randomly divided six healthy heifers into two groups. The first group received a control diet, whereas the second group received Cd-exceeding diets for 100 days. After 100 days, the livers were collected. A total of 551 differentially expressed mRNAs, 24 differentially expressed miRNAs, and 169 differentially expressed lncRNAs were identified (*p* < 0.05, |log2FC| >1). Differentially expressed genes (DEGs) were analyzed by gene ontology and Kyoto Encyclopedia of Genes and Genomes enrichment analyses. We found that under Cd exposure, DEGs were enriched in the adenosine 5'-monophosphate–activated protein kinase pathway, which is involved in autophagy regulation, and the peroxisome proliferator–activated receptor pathway, which is involved in lipid metabolism. In addition, the apolipoprotein A4 gene, which has anti-inflammatory and antioxidant effects, the anti-apoptotic gene ATPase H^+^/K^+^ transporting the nongastric alpha2 subunit, and the cholesterol metabolism–associated gene endothelial lipase gene were significantly downregulated. C–X–C motif chemokine ligand 3, cholesterol 7α-hydroxylase, and stearoyl-CoA desaturase, which are involved in the development of fatty liver, were significantly upregulated. These genes revealed the main effects of Cd on the liver of Xiangxi yellow heifers. The current study provides insightful information regarding the DEGs involved in autophagy regulation, apoptosis, lipid metabolism, anti-inflammation, and antioxidant enzyme activity. These may serve as useful biomarkers for predicting and treating Cd-related diseases in the future.

## Introduction

Heavy metals accumulate significantly in the soils of most parts of China, with cadmium (Cd) being the most prevalent heavy metal contaminant. Contaminated areas are mainly located in the central, southern, and southwestern parts of China, and Hunan is one of the most seriously contaminated provinces (1). There are several large mines and smelting areas in the Hunan Province, which is known for its non–ferrous metal mining activities and is a typical area of dense low-temperature deposits in China (2). Heavy metal contamination in soil is considered a potential cancer-causing health risk for Hunan residents. Moreover, heavy metal pollution in rivers used for agricultural irrigation in Hunan Province is considered to indirectly affect the health of humans and animals (3). Garden soil, rice soil, vegetables, and rice in the Hunan mining area are contaminated with heavy metals (4). Cd pollution is considered to be the most serious heavy metal pollution in Hunan. The Cd content in both rice straw and endosperm samples in Hunan is higher than that in the Yangtze River delta (5). The western part of Hunan is known as Xiangxi. The Xiangxi yellow heifer is the main farmed animal in the Xiangxi region, which mainly consumes local forage. This is highly significant for the development of the Xiangxi farming industry. The beneficial characteristics of Xiangxi yellow heifers include mild temperament, resistance to heat, medium size, well-developed and strong bones, tender meat, and rich nutrition (6). Beef is one of the main food sources for the local people. Studies have shown that metal accumulation in livestock can be transferred to humans through the food chain (7, 8).

Heavy metals greatly endanger the health of animals and humans through environmental exposure and the food chain. Cd contamination has caused several vicious incidents, including the Cd rice incident in Hunan, China, and the Itai-Itai disease public health incident in Japan. In China, Hunan, Henan, Anhui, and Jilin provinces have high levels of Cd in the soil. Cd in Hunan farmland exceeds the soil environmental background value by 30-fold (9). Cd is absorbed mainly by the respiratory and digestive tracts. Moreover, Cd adversely affects the liver, kidneys, lungs, immune system, reproductive system, and cardiovascular system (10). When Cd-containing foods and water are mistakenly consumed, Cd is transported from the intestine to the liver, causing functional disorders (11). Studies have shown that Cd interferes with collagen metabolism and extracellular signal transduction in the liver matrix, as well as cell adhesion, growth, and migration (12–14). Cd also causes morphological and structural changes in the liver, such as blurred hepatic trabeculae, increased cell size, hepatocyte deformation, nuclear consolidation, lipid droplet aggregation, and mono-nuclear cell infiltration (15). Hepatic mitochondrial function has been reported to be impaired in a rat model of Cd exposure (16–18). Cd exposure inhibits mitochondrial oxidative phosphorylation, resulting in a decrease in ATP. In addition, high concentrations of Cd inhibit basal respiration (19). Moreover, Cd can directly damage the mitochondrial structures in the liver, causing mitochondrial degeneration, vacuolation, and disruption of the mitochondrial membrane structure (20, 21). In the presence of severe mitochondrial damage, the process of mitochondrial autophagy is activated. Metallothionein (MT), which binds to Cd, protects organisms from Cd toxicity. Cd decreases the levels of MT and antioxidant glutathione (GSH) and inhibits the activity of antioxidant enzymes (22). Therefore, it can induce oxidative stress (23, 24). Cd can also induce systemic inflammatory responses involving innate, adaptive, and mucosal immunity (25). In addition, the increase in Ca^2+^ levels accelerates reactive oxygen species (ROS) formation, also resulting in oxidative stress (26, 27). Cd induces endoplasmic reticulum stress and DNA mutations by inhibiting the DNA repair system and inducing protein damage (28–30). Cd damages the adenosine triphosphate system and the tricarboxylic acid cycle, which can lead to an increase in various metabolites related to lipid metabolism and triglycerides and disrupted energy metabolism (18). Cd exposure is a potential risk factor for the development of diabetes mellitus, non-alcoholic steatohepatitis (NASH), and non-alcoholic fatty liver disease (NAFLD). Cd exacerbates hepatocyte lipid storage through dysregulation of autophagy, which aggravates hepatic steatosis and leads to hyperlipidemia (31). Cd is also associated with an increased risk of prostate, breast, lung, and hepatocellular carcinoma (32–34).

RNA-seq, characterized by low background noise and a large detection range, can provide a large amount of information on gene expression levels, facilitating the study of genetic mechanisms. Currently, it is the preferred method for studying gene expression (35, 36). Some studies have claimed that heifers are potential biological carriers of heavy metal contamination in soil (8). An association exists between metal levels in heifer tissues and the risk of human exposure. In addition, the liver is the most prominent target organ of Cd toxicity (37).

Therefore, in this study, the Hunan landmark product Xiangxi yellow heifers were used as experimental animals. Then, RNA-seq was used to study the effects of feeding Cd overload diets on the expression of mRNAs, miRNAs, and lncRNAs in the liver of Xiangxi yellow heifers to understand their transcriptional biology. At present, there are numerous studies on the hepatotoxicity of Cd, but there is a relative lack of information on the hepatic transcriptomics of Cd-exposed Chinese Xiangxi yellow heifers. Hunan Province is rich in mineral resources. However, Cd pollution is present. Cd has great potential to endanger the health of the local dominant breed of Xiangxi yellow heifers. Therefore, this study hypothesized that certain transcription factors and metabolic pathways would be affected by Cd exposure during foraging in Chinese Xiangxi yellow heifers. The current experiment aimed to find the pathological changes that occurred in the liver of Xiangxi yellow cattle after feeding Cd-containing forage. This experiment also aimed to analyze the gene expression at the RNA level and the pathways that are significantly enriched in Xiangxi yellow cattle exposed to cadmium using transcriptomics. Our findings provide new insights into the potential health risks of Cd, improve the current understanding of the toxic effects of Cd on the liver, and help to prevent Cd toxicity.

## Materials and Methods

### Materials, Animals, and Treatment

The experiments were conducted at the National Origin Farm of Xiangxi Cattle, which is located 10 km south of Huayuan County, Hunan, China. All experimental protocols and procedures used in this study followed Chinese standards for the use and care of research animals (SOP-017).

A total of six healthy female crossbred (Angus bull × Xiangxi local cattle) heifers weaned (BW = 78.50 ± 18.45 kg) at 6 months of age were selected from a paternal halfsib family. They were randomly allocated to the control group (control) or cadmium-treated group (cadmium), with three heifers in each group. Heifers were housed in an open-sided, naturally well-ventilated barn, and the groups were separated using railings. Thus, three heifers within each group were housed together in one pen. The feeding experiments were conducted for 109 days. The first 9 days were the pre-feeding period, during which the test heifers were dewormed and acclimatized to the diet. The following 100 days were the regular experimental feeding periods. During the experiments, the diets of both groups consisted of concentrate, paddy, and corn straw silage (Table 1). These diets were mixed daily and provided to the cattle a total mixed ration. In China, the standard for agricultural soil cadmium is 0.3 mg/kg (38). The corn silage used for the cadmium-treated group was made from a whole-plant corn species with strong cadmium enrichment ability (Yuqing 23). This corn was planted in soil with excessive cadmium content (3–5 mg/kg) in Yonghe Town, Liuyang City, Hunan Province. The corn silage used for the control group was made from normal whole-plant corn that was planted in soil with a Cd content of 0.2 mg/kg. The Cd concentrations of the diets in the control and cadmium groups were 0.72 and 6.74 mg/kg, respectively. The orts were removed one time daily before the morning feed, and a new feed was delivered two times daily at 7:00 a.m. and 17:00 a.m. Cattle within each group shared one feed trough. All the cattle had free access to water. A part of the liver was placed in 10% formalin and used to prepare histological sections for morphological analysis. The remaining liver tissue samples were frozen at a −80°C in a refrigerator for RNA-seq.

**Table 1:** Feed formulation for the control group and cadmium-treated group.

**Feed indicators**	**Control**	**Cadmium**
Concentrate (%)	11.34	11.34
Paddy (%)	2.84	2.84
Corn straw silage (%)	85.82	85.82
**Nutritive index**		
Gross energy (MJ/kg)	13.36	12
Crude protein (%)	10.79	11.45
Crude fat (%)	10.77	12.65
CF (%)	11	13
NDF (%)	49.33	50.67
ADF (%)	25	25.33
Ca (%)	0.62	0.62
P (%)	0.42	0.37
Cd (mg/kg)	0.73	6.07

### Preparation of Liver Tissue Sections

Liver tissues were fixed in 10% formalin for 24–48 h, followed by dehydration in different grades of ethanol. Next, 6-μm-thick sections were cut from the prepared liver blocks, affixed to glass slides, rehydrated, and stained with hematoxylin and eosin. The prepared slides were observed under a microscope equipped with a microphotographic system.

### Measuring Cd Concentration in Liver Tissues Using Atomic Absorption Spectrophotometry

Different concentrations (0, 0.1, 0.2, 0.4, 0.6, 1.0, 2.0, 3.0, and 4.0 μg/mL) of HNO_3_ solution were prepared by the National Center for Analysis and Testing of Non-ferrous Metals and Electronic Materials.

First, 0.5 g of the liver was dried in a desiccator at 75°C for 1 h. Then, 3 mL of the digestion solution (HNO_3_:HCLO = 7:3) was added and heated to near evaporation. The residue was dissolved in 1.0% HNO_3_. The samples to be measured and cadmium standard solutions with different standard concentrations were placed in the Z-5000 graphite furnace atomic absorption spectrophotometer. Finally, the determination was carried out separately according to a pre-set procedure (drying at 80–140°C for 40 s; ashing at 300°C for 20 s; and atomization at 1,500°C for 5 s). Finally, a concentration–absorbance curve was automatically generated by the machine.

### Library Preparation and Sequencing

Liver tissue samples frozen at −80°C were used for RNA-seq. A 50–100 mg sample was collected from each group and added to 0.5 ml TRIzol (Invitrogen, Carlsbad, CA, United States) on ice. Then, the samples were mixed using a homogenizer (Cebo, Shanghai, China), and 0.5 mL TRIzol was added. The tissue and TRIzol were transferred together into a 1.5 mL EP tube and left at 37°C for 5 min. The sample was centrifuged at 12,000 × g for 5 min and the sediment was discarded. Then, 0.2 mL of chloroform was added. The samples were shaken for 15 s and incubated at room temperature for 3 s. The samples were then centrifuged at 4°C for 20 min at 12,000 × g. When the samples appeared to be stratified, the RNA was mainly in the upper aqueous phase; therefore, the aqueous phase was transferred to a new tube. Immediately after, 100% isopropanol (0.5 mL) was added. The samples were mixed by inversion and left at 37°C for 10 min. The supernatant was removed by centrifugation at 12,000 × g for 10 min at 4°C. Then, 1 mL of 80% ethanol was added and the sample was mixed. After centrifugation at 7,500 × g for 5 min at 4°C, the supernatant was discarded. The samples were then left at room temperature for approximately 5–10 min. Finally, 50 μL of DEPC water was added to solubilize the RNA, and the RNA solution was obtained by centrifugation under a light flow. Total RNA concentration and quality were determined using a NanoDrop spectrophotometer and an Agilent 2,100 Bioanalyzer (Thermo Fisher Scientific, MA, United States).

Two new libraries, lncRNA/mRNA and miRNA, were constructed. In the lncRNA/mRNA library, the Ribo-Zero Magnetic Kit (Epicenter) was used to remove 5.8S rRNA, 18S rRNA, 28S rRNA, 12S rRNA, and 16S rRNA from total RNA. Single-stranded cDNA was synthesized using random primers and reverse transcriptase. DNA polymerase I and RNase H (Invitrogen) were used to synthesize the second-strand cDNA. At this point, the RNA template was removed and dTTP was replaced with dUTP. The double-stranded cDNA product was then subjected to 3'-adenylation and splice ligation. The products were subjected to several rounds of PCR and thermal denaturation to a single strand and then cyclized by splint oligo to obtain single-stranded circular DNA. Paired-end sequencing (100 bp) was performed using a BGISEQ-500/MGISEQ-2000 system (BGI-Shenzhen, China). Each sample from the lncRNA/mRNA library yielded an average of 13.65 G of data.

For miRNA sequencing, 18–30 nt of small RNA (Ladder Marker, Takara) was isolated and purified from total RNA by polyacrylamide gel electrophoresis (PAGE) gel cutting. The 5-adenylated, 3-blocked single-stranded DNA junction was ligated to the 3' end of the purified RNA, and the 5' junction was ligated to the 5' end of the RNA. RNA with 3' and 5' junctions attached was synthesized into cDNA by reverse transcription extension using RT primers bearing UMI tags. The cDNA was subsequently amplified by PCR. The PCR products were separated by PAGE to obtain fragments in the range of 110–130 bp. Fragments were purified using a QIAquick Gel Extraction Kit (QIAGEN, CA, United States) and quality-controlled using an Agilent 2,100 Bioanalyzer (Thermo Fisher Scientific, United States). The purified library products were subsequently sequenced at the 50 bp single-end on the DNBseq platform (BGI-Shenzhen, China). The raw data obtained from sequencing were converted into raw sequence data (raw data or raw reads) by base calling and stored in the fastq file format, which contains the sequence of reads and sequencing quality information. The miRNA library yielded an average of 23.77 M data per sample. miRanda (3.3a) and TargetScan (7.1) were used to predict the target genes of the miRNAs, and the David database (6.8) was used for pathway enrichment analysis of the miRNA target genes.

### Transcriptome Data Analysis

The sequencing data in the fastq format were filtered using SOAPnuke (v1.5.2) (39) for reads with unknown base N content >5%, reads with splice contamination, and reads without insert tags. The clean reads obtained were then mapped to the reference genome *Bos taurus* (vGCF_000003205.7_Btau_5.0.1) using Bowtie2 (v2.2.5) (40). mRNA expression was calculated using RSEM (v1. 2.12), which uses the normalization method fragments per kilobase of exon model per million mapped fragments (FPKM). miRNA expression was calculated using reads of exon model per million mapped reads values. Finally, the DESeq2 (41) method was used to determine the differential expression of two groups of genes (three biological replicates per group) based on a negative binomial distribution model with high sensitivity and accuracy. The obtained *P* was calculated using the likelihood-ratio test to represent the difference between the two groups of samples at the inclusion level. For DEG analysis, a *P* ≤ 0.05 and | log2FC |>1 were considered significant.

### Gene Ontology and Kyoto Encyclopedia of Genes and Genomes Enrichment Analyses

To annotate the biological functions of the genes, all differentially expressed mRNAs (DEmRNAs) were aligned against the KEGG and GO databases. The number of genes per GO term and the enriched KEGG pathways were calculated. The candidate genes were then compared to all background genes of the species using the phyper function (https://en.wikipedia.org/wiki/Hypergeometric_distribution) based on a hypergeometric test to obtain the *p*. The *p*-values were corrected using the Benjamini–Hochberg algorithm to obtain FDR values. FDR ≤ 0.05 was used as the threshold value. Finally, the GO terms and KEGG pathways that met this condition were recognized as significantly enriched compared with the background genes of this species.

In the histogram of GO enrichment, the *X*-axis represents the number of genes annotated to a particular term. The *Y*-axis represents the number of selected genes annotated to a particular term. In the KEGG pathway diagram, the rich factor on the *X*-axis represents the ratio of the number of DEGs in the metabolic pathway to the number of genes annotated in the pathway, with larger values indicating greater enrichment. The *Y*-axis represents the pathway to which the gene was annotated. Box plots and variable shear plots were constructed to determine the quality of the RNA-seq data. The box plot demonstrates the distribution of gene expression levels for each sample and displays the dispersion of the data distribution, which was generated using the OmicStudio tools (https://www.omicstudio.cn/tool). rMATS (v3.2.5) (42) was used to detect splicing events in the samples.

### RNA Extraction

Liver tissues of Xiangxi yellow heifers were removed from the −80°C refrigerator. Total RNA was extracted according to the instructions. A small portion of the liver was homogenized in 1 mL of TRIzol. Then, 200 μL of chloroform was added and the sample was shaken vigorously. The sample was centrifuged at 1,200 × g for 3 min and the supernatant was transferred to an absorption column. Next, 400 μL of 96% ethanol was added and recentrifuged at 12,000 × g for 1 min. The supernatant was removed from the absorption column and 500 μL of 3 M sodium acetate trihydrate solution was added. It was then subjected to centrifugation at 12,000 × g for 1 min. Finally, 70% alcohol (400 μL) was added and the mixture was centrifuged at 12,000 × g in triplicate. The supernatant was removed from the absorption column, and 46–50 μL of DEPC was added to the RNA pellet. Total RNA was collected in Eppendorf tubes and centrifuged for 1 min at 10,000 × g. Total RNA content was measured using a NanoDrop spectrophotometer and an Agilent 2,100 Bioanalyzer (Thermo Fisher Scientific, MA, United States).

### Quantitative RT–PCR

cDNA was synthesized by reverse transcription using the Superscript III reverse transcription kit (ABI Invitrogen, United States). qRT-PCR was performed for genes associated with Cd exposure using a fluorescent quantitative PCR instrument (Applied Biosystems, United States). Specific quantitative primers (KuMei, Changchun, China) for the 18 transcripts are shown in Supplementary Table S1. The total volume of the qRT-PCR mixture was 20 μL, which contained 10 μL of SYBR (Takara Bio), 1 μL of the forward primer, 1 μL of the reverse primer, 2 μL of the cDNA, and 6 μL of ddH_2_O. The reaction conditions were as follows: 1 cycle at 95°C for 5 min, 40 cycles at 95°C for 10 s, 58°C for 20 s, 72°C for 20 s, and 1 cycle at 95°C for 15 s. The magnitude of the fold differences in gene expression levels was calculated using the 2^−ΔΔCT^ method. An independent-samples *t*-test was used to test the difference between the cadmium-treated and control group. Statistical significance was set at *p* < 0.05.

### Statistical Analysis

*t*-Tests were performed on all experimental data in triplicate using IBM SPSS Statistics 22 and GraphPad Prism 5. All results are expressed as mean ± SEM, and the significance level was set at ^*^ *p* < 0.05, ^**^ *p* < 0.01, and ^***^ *p* < 0.001.

## Results

### Cadmium Accumulation and Pathological Changes in the Liver

**Figure 1:**
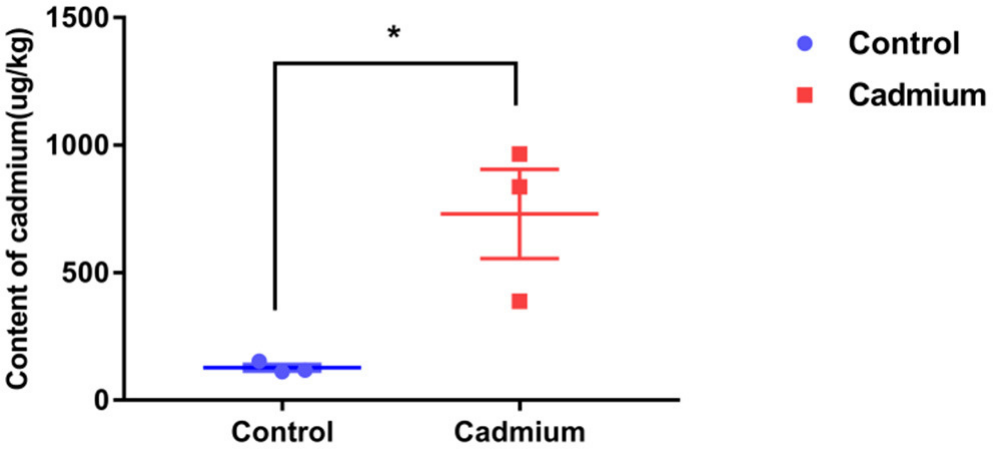
The content of cadmium in liver tissues. Cadmium content in the liver tissues of the control group (Control) and cadmium-treated group (Cadmium), as obtained by graphite furnace atomic absorption spectrophotometry (μg/kg). Data were analyzed by independent samples *t*-test. The significance level was set at * *p* < 0.05.

The Cd content in the liver of heifers in the Cd-treated group was significantly higher than that in the control group. This finding indicates that Cd accumulated in the liver of Xiangxi yellow heifers fed a high-Cd diet for 100 days (Figure 1).

**Figure 2:**
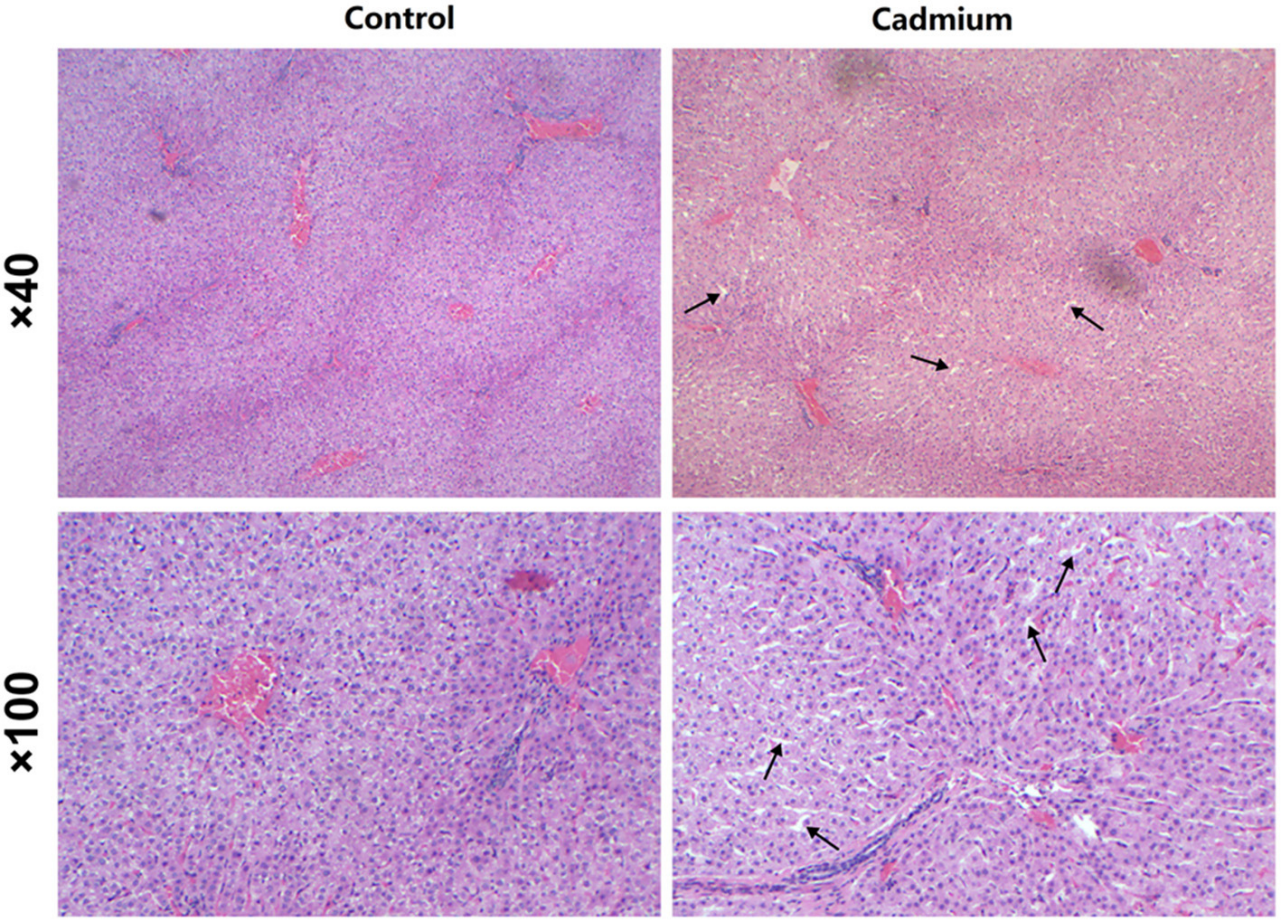
H and E staining. Pathological changes in the liver of control heifers (Control) and cadmium-treated heifers (Cadmium) by H and E staining (40×, 100×). Arrows indicate expanded hepatic sinusoids.

Histopathological observations reveal that the liver of heifers in the cadmium-treated group had expanded sinusoids compared to the control group (Figure 2).

### Analysis of RNA-seq Libraries

To evaluate the toxic effects of Cd on the liver of Xiangxi yellow heifer, the liver was collected for whole transcriptome analysis. As shown in Table 2, a total of 132–140 million raw reads were obtained. After removing reads with an unknown base N content >5% and low-quality reads, 131–141 million clean reads remained, and 13–14 Gb of clean bases were acquired.

**Table 2:** Data filtering for RNA-seq.

**Sample**	**Total raw reads (M)**	**Total clean reads (M)**	**Total clean bases (Gb)**	**Clean reads Q20 (%)**	**Clean reads Q30 (%)**	**Clean reads ratio (%)**
Control1	139.93	138.24	13.82	98.37	95.21	98.79
Control 2	134.93	134.10	13.41	98.36	95.07	99.39
Control 3	142.43	141.00	14.10	98.07	94.27	99.00
Cadmium 1	132.97	131.50	13.15	98.09	94.38	98.89
Cadmium 2	137.43	136.61	13.66	98.34	95.03	99.40
Cadmium 3	134.93	133.94	13.39	98.57	95.73	99.27

*Reads filtering: Raw Reads: number of reads before filtering (million), Clean Reads (M): number of reads after filtering (million), Clean Bases (Gb): number of bases after filtering (Gb), Clean ReadsQ20 (%): quality of reads after filtering >Q20 ratio, Clean Reads Q30 (%): quality of filtered reads >Q30 ratio, Clean Reads Ratio (%): ratio of filtered reads*.

**Figure 3:**
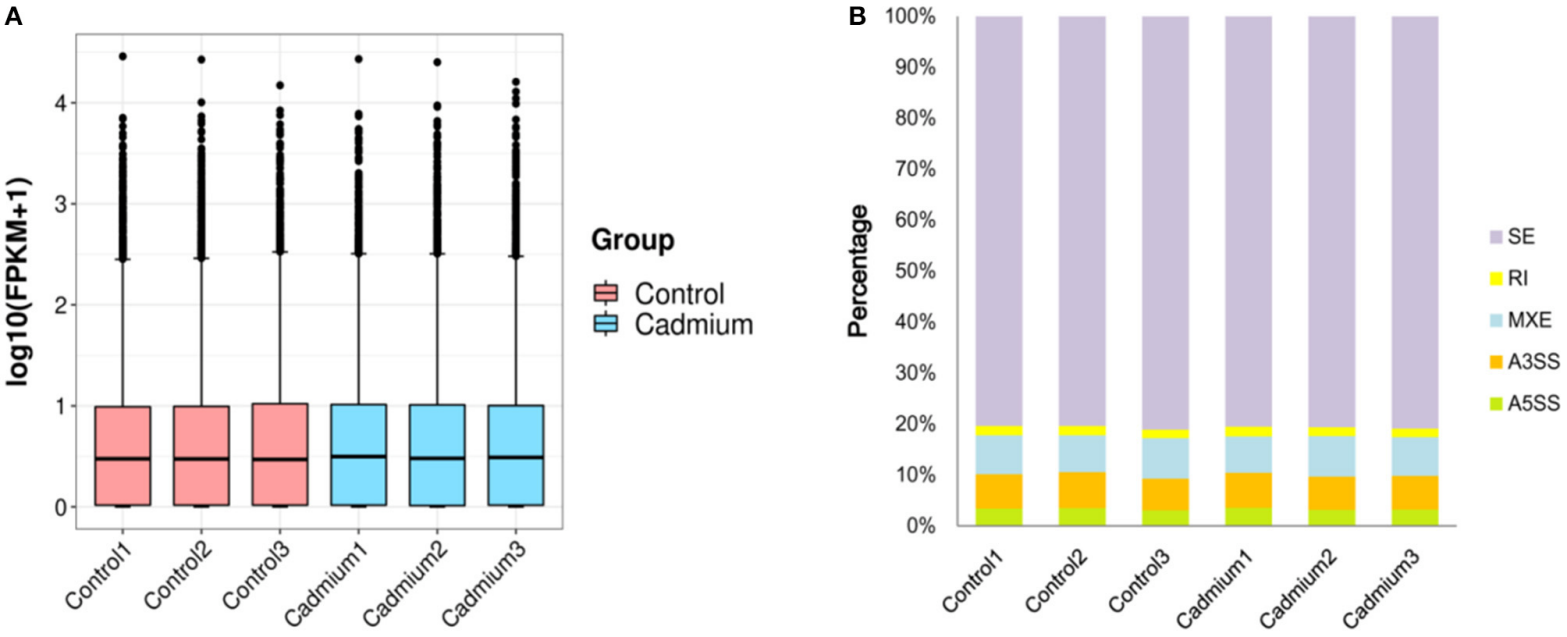
Box plots and variable shear events. **(A)** Box plots of the gene expression levels: the *X*-axis is the sample name, the *Y*-axis is log10 (FPKM+1), and each region of the box plot corresponds to five statistics (upper limit, upper quartile, median, lower quartile, and lower limit, respectively, from top to bottom, where upper and lower limits are not considered outliers). **(B)** Variable Shear Events: alternative 5' splicing site (A5SS), alternative 3' splicing site (A3SS), mutually exclusive exons (MXE), retained intron (RI), skipped exon (SE).

Clean reads were mapped to the reference genome. The localization rate was ~91.8% (Table 3). Approximately 88.6% of the reads could be uniquely mapped to the *Bos taurus* genome. The box plot represents the degree of dispersion and volatility of the data distribution (Figure 3A). Figure 3A shows that the boxes were symmetrical about the middle line, indicating that the data were normally distributed. The smaller height of the boxes was reflected by the smaller degree of fluctuation in the data. Good standardization of the data was indicated by the consistency of the data distribution of the samples in Figure 3A. As shown in Figure 3, mRNA was mainly generated by exon skipping to produce splice isoforms of different mRNAs and regulate gene expression.

**Table 3:** Reference genome mapping.

**Sample**	**Total clean reads (M)**	**Total mapping(%)**	**Uniquely mapping(%)**
Control 1	138.24	92.55	88.93
Control 2	134.1	92.15	88.91
Control 3	141	91.26	88.44
Cadmium 1	131.5	90.57	88.11
Cadmium 2	136.61	92.07	88.47
Cadmium 3	133.94	91.9	88.07

*The total mapping ratio is the overall mapping ratio of the sequenced reads to the reference genome and the uniquely mapping ratio is the mapping ratio of the sequenced reads to the reference genome*.

### DEGs Analysis

**Figure 4:**
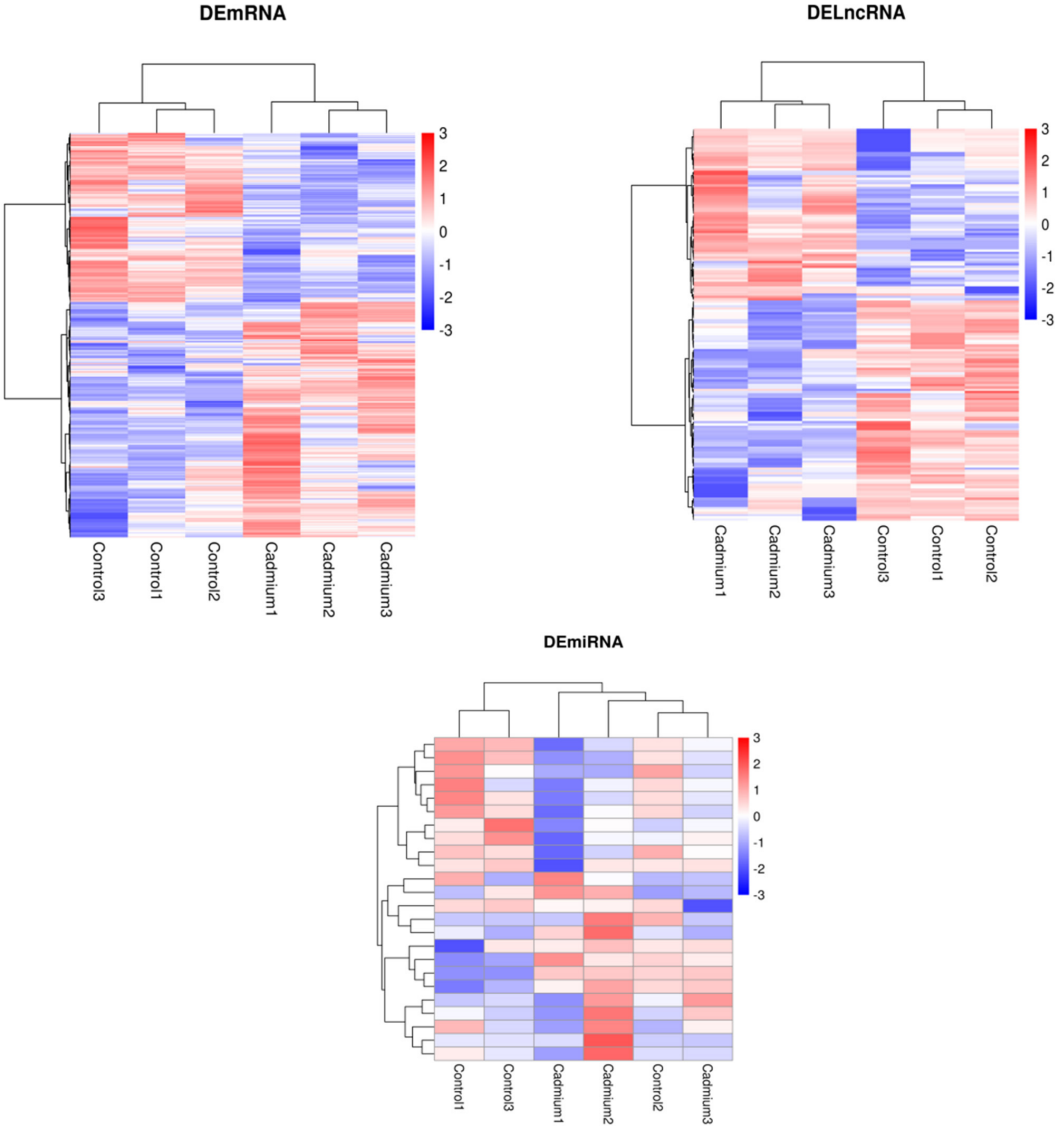
Heatmap of DEmRNAs, DElncRNAs, and DEmiRNAs clustering analysis. Red represents upregulated genes, and blue represents downregulated genes.

Cluster analysis of all DEGs was performed to observe gene expression patterns. Heat maps were generated, with red and blue representing upregulated and downregulated genes, respectively (Figure 4). The overall expression pattern of genes in the Cd-treated group was quite different from that of the control group, and the three samples within the same group showed similar expression patterns. This indicates that the samples from both groups were reproducible, demonstrating that the derived data were credible.

**Figure 5:**
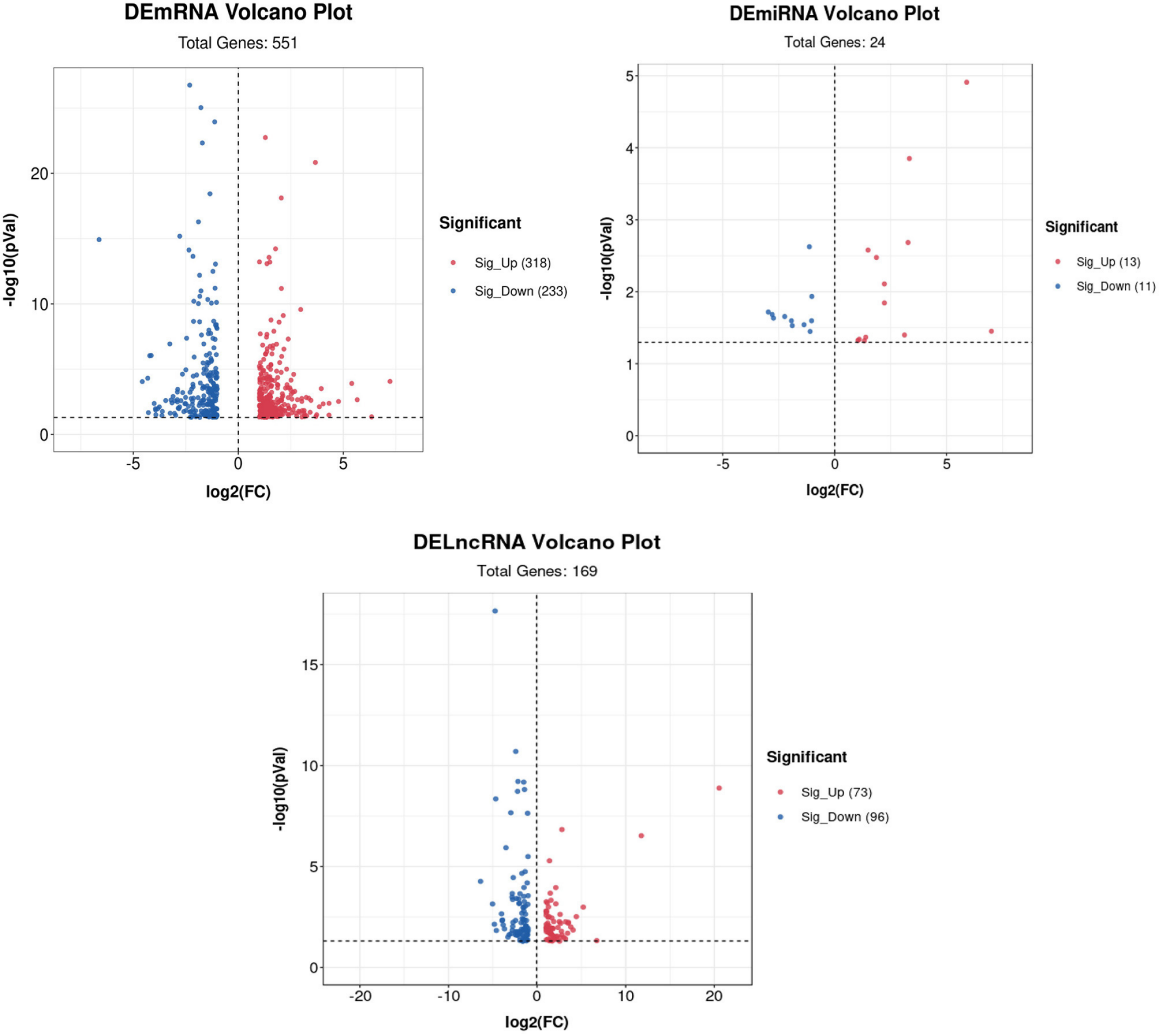
Volcano plot of DEmRNAs, DElncRNAs, and DEmiRNAs. Red represents upregulated genes, and blue represents downregulated genes.

Volcano plots of the DEGs showed the differences in the distribution of gene expression levels between control and Cd-treated samples. A total of 551 DEmRNAs were identified in the control and cadmium-treated bovine livers. Of these, 318 genes were upregulated, and 233 genes were downregulated. Compared with the control group, 24 differentially expressed miRNAs (DEmiRNAs) were identified. Of these, 13 genes were upregulated and 11 downregulated in the cadmium-treated group. In total, 169 differentially expressed lncRNAs (DElncRNAs), were identified. Of these, 73 genes were upregulated and 96 genes downregulated (Figure 5).

### Functional Analyses of DEGs

**Figure 6:**
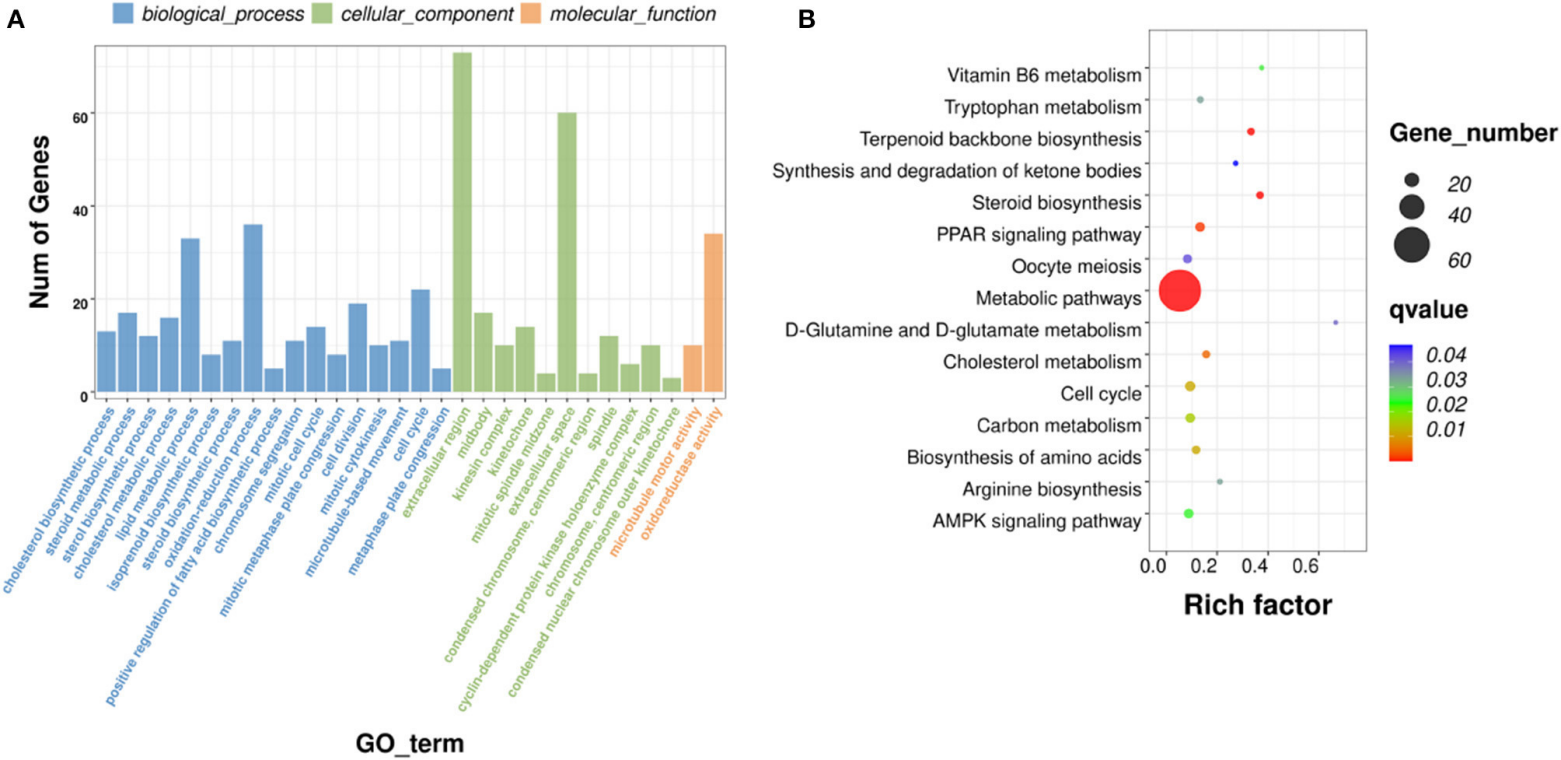
GO and KEGG pathway analyses. **(A)** Histograms of GO enrichment, KEGG functional classification of DEmRNAs. **(B)** Significantly enriched KEGG pathways (padj <0.05).

A total of 551 significant DEGs in the liver, were subjected to GO and KEGG pathway analyses to determine the enrichment of functions and action pathways by DEGs. Based on the results of the functional classification of DEGs in the categories of biological process (BP), cellular component (CC), and molecular function (MF), 92 GO terms were obtained. The terms of interest were included in the first 30 enriched GO terms (including oxidation–reduction process, cholesterol biosynthetic process, cell cycle, cyclin B1–CDK1 complex, cyclin-dependent protein kinase holoenzyme complex, and oxidoreductase activity) (Figure 6A). Table 4 lists the DEmRNAs involved in these functionally related events.

**Table 4:** DEGs included in the GO terms.

**Gene ID**	**Gene name**	**log2FC**	***P*-value**
**Cell cycle**			
528138	KLHL13	−1.03	6.21E-09
509117	SPC24	2.32	1.53E-04
786629	E2F8	2.50	3.96E-04
414925	BIRC5	2.13	1.10E-03
327679	CCNB1	2.08	1.51E-03
540455	PIMREG	3.02	1.76E-03
505200	CCNF	1.87	2.05E-03
524530	CCND1	1.14	2.27E-03
281061	CDK1	1.89	3.67E-03
534849	ASPM	1.59	9.13E-03
515249	CKAP2	1.38	9.50E-03
617456	HJURP	1.80	9.79E-03
281668	CCNB2	1.33	1.95E-02
527124	CDKN2B	−1.19	2.02E-02
615282	CDKN3	2.93	2.04E-02
529478	SYCE1	1.80	2.08E-02
540206	MASTL	1.32	2.75E-02
510809	MIS18A	2.05	2.93E-02
360192	AURKB	1.05	3.69E-02
526129	E2F7	1.34	4.40E-02
616028	NUSAP1	1.24	4.48E-02
281667	CCNA2	1.22	4.90E-02
**Cholesterol biosynthetic process**			
281156	FDPS	1.61	6.71E-07
281631	APOA1	−2.79	6.57E-16
281767	FDFT1	1.17	1.22E-04
407159	HMGCR	2.14	7.78E-10
503684	HMGCS2	−1.08	2.83E-05
505060	CYP51A1	1.83	1.22E-07
505792	MVK	1.21	1.25E-05
509958	MVD	1.30	7.23E-07
514293	IDI1	1.16	1.44E-07
514745	DHCR7	1.55	4.42E-06
533726	DHCR24	1.54	1.03E-05
537301	APOA4	−4.15	8.99E-07
615906	LSS	1.36	2.16E-08
**Cyclin B1–CDK1 complex**			
281061	CDK1	1.89	3.67E-03
327679	CCNB1	2.08	1.51E-03
**Cyclin-dependent protein kinase holoenzyme complex**			
281061	CDK1	1.890651445	0.003667074
281667	CCNA2	1.221442141	0.048959244
281668	CCNB2	1.33210537	0.019457532
327679	CCNB1	2.080892781	0.001514292
505200	CCNF	1.865651842	0.002046151
524530	CCND1	1.140768699	0.002273159
**Oxidoreductase activity**			
280844	LPO	−3.94	1.08E-02
280924	SCD	2.97	2.68E-10
281152	FASN	1.43	3.72E-05
281274	LDHA	−1.63	9.58E-06
281275	LDHB	−1.37	3.06E-04
281785	GLUD1	1.01	6.07E-14
281904	LOXL4	1.44	7.06E-06
282022	PTGS1	1.12	6.45E-03
282870	CYP1A1	1.27	5.94E-03
338074	AOX1	1.49	4.94E-05
404131	HSDL2	−1.08	8.96E-14
407159	HMGCR	2.14	7.78E-10
504481	MSMO1	1.69	1.24E-08
505060	CYP51A1	1.83	1.22E-07
508167	RRM2	1.26	1.09E-02
508591	MIOX	−1.50	4.03E-02
510507	CYP7A1	2.40	3.40E-02
511470	CYP1B1	1.31	2.87E-03
513221	HMOX1	1.22	3.31E-03
514745	DHCR7	1.55	4.42E-06
515710	HSD17B2	1.14	2.40E-03
516036	CBR3	−1.17	1.22E-02
516864	ALDH1L2	1.19	6.53E-03
518393	AOX4	5.40	1.25E-04
526295	AIFM3	−3.04	2.43E-02
533088	GPX2	2.12	2.82E-02
533107	FADS1	1.97	3.13E-06
533726	DHCR24	1.54	1.03E-05
534041	MICAL2	−1.07	1.84E-03
534599	OGDH	−1.32	1.85E-05
539606	PYCR1	1.45	4.18E-03
540994	DEGS2	1.40	2.87E-04
617564	DHDH	1.60	4.44E-02
788719	FMO5	1.02	6.62E-04
**Oxidation–reduction process**			
280844	LPO	−3.94	1.08E-02
281152	FASN	1.43	3.72E-05
281274	LDHA	−1.63	9.58E-06
281275	LDHB	−1.37	3.06E-04
281785	GLUD1	1.01	6.07E-14
281904	LOXL4	1.44	7.06E-06
282022	PTGS1	1.12	6.45E-03
282870	CYP1A1	1.27	5.94E-03
338074	AOX1	1.49	4.94E-05
404131	HSDL2	−1.08	8.96E-14
407159	HMGCR	2.14	7.78E-10
504481	MSMO1	1.69	1.24E-08
505060	CYP51A1	1.83	1.22E-07
508167	RRM2	1.26	1.09E-02
508591	MIOX	−1.50	4.03E-02
510507	CYP7A1	2.40	3.40E-02
511470	CYP1B1	1.31	2.87E-03
513221	HMOX1	1.22	3.31E-03
514361	ERO1B	−1.19	3.94E-04
514745	DHCR7	1.55	4.42E-06
515710	HSD17B2	1.14	2.40E-03
516036	CBR3	−1.17	1.22E-02
516864	ALDH1L2	1.19	6.53E-03
518393	AOX4	5.40	1.25E-04
526295	AIFM3	−3.04	2.43E-02
526535	SQLE	3.67	1.47E-21
533088	GPX2	2.12	2.82E-02
533107	FADS1	1.97	3.13E-06
533726	DHCR24	1.54	1.03E-05
534041	MICAL2	−1.07	1.84E-03
534599	OGDH	−1.32	1.85E-05
539606	PYCR1	1.45	4.18E-03
540994	DEGS2	1.40	2.87E-04
617564	DHDH	1.60	4.44E-02
618192	HSD17B13	−1.19	1.19E-04
788719	FMO5	1.02	6.62E-04

**Figure 7:**
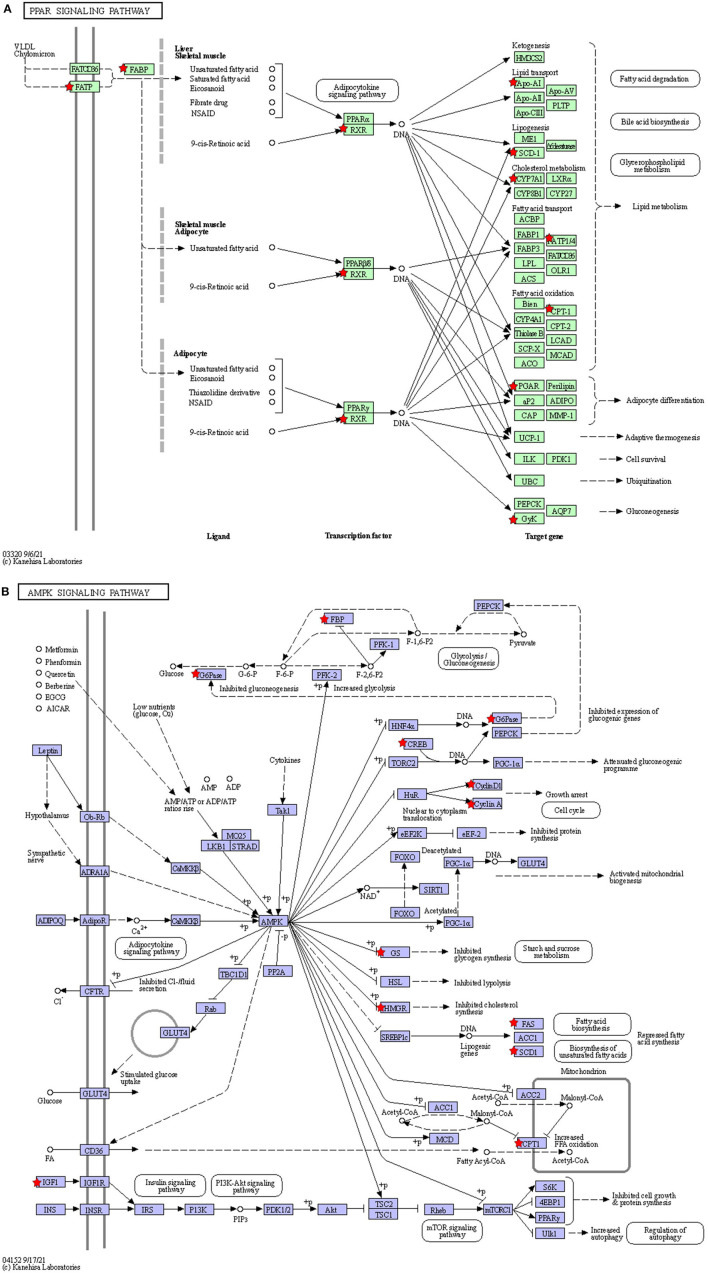
The location of the DEGmRNAs in the significantly enriched pathways. Diagram of the PPAR signaling pathway **(A)** and AMPK signaling pathway **(B)**. The red pentagram represents DEGmRNAs.

The KEGG pathways that were significantly enriched (padj <0.05) were metabolic, peroxisome proliferator–activated receptor (PPAR) signaling, AMPK signaling, and steroid biosynthesis pathways (Figure 6B; Table 5). Table 6 shows the DEmRNAs involved in these signaling pathways in Cd-exposed livers of Xiangxi yellow heifers and the positions of these DEmRNAs in the pathways (Figure 7).

**Table 5:** Results of the KEGG enrichment analysis of DEGs.

**Pathway ID**	**KEGG pathway**	**Rich ratio**	**Gene number**	***Q*-value**
ko01100	Metabolic pathways	5.31E-02	72	2.54E-05
ko00100	Steroid biosynthesis	3.68E-01	7	6.56E-05
ko00900	Terpenoid backbone biosynthesis	3.33E-01	7	9.59E-05
ko03320	PPAR signaling pathway	1.33E-01	11	1.42E-03
ko04979	Cholesterol metabolism	1.57E-01	8	4.29E-03
ko04110	Cell cycle	9.38E-02	12	1.15E-02
ko01230	Biosynthesis of amino acids	1.17E-01	9	1.15E-02
ko01200	Carbon metabolism	9.40E-02	11	1.59E-02
ko04152	AMPK signaling pathway	8.80E-02	11	2.45E-02
ko00750	Vitamin B6 metabolism	3.75E-01	3	2.55E-02
ko00380	Tryptophan metabolism	1.33E-01	6	3.36E-02
ko00220	Arginine biosynthesis	2.11E-01	4	3.36E-02
ko00471	D-Glutamine and D-glutamate metabolism	6.67E-01	2	3.94E-02
ko04114	Oocyte meiosis	8.26E-02	10	4.20E-02
ko00072	Synthesis and degradation of ketone bodies	2.73E-01	3	4.68E-02

**Table 6:** DEGs included in the PPAR signaling pathway, metabolic pathways, and AMPK signaling pathway.

**Gene ID**	**Gene name**	**log2FC**	***P*-value**
**PPAR signaling pathway**			
107131384	PLIN4	−1.32	9.90E-04
280924	SCD	2.97	2.68E-10
281631	APOA1	−2.79	6.57E-16
281760	FABP5	−1.32	4.83E-03
505987	GK	−1.68	3.33E-06
509459	CPT1B	−1.16	2.88E-03
509500	RXRG	−1.84	2.36E-09
509963	ANGPTL4	−1.84	6.41E-13
510507	CYP7A1	2.40	3.40E-02
513787	SLC27A1	−2.12	1.89E-02
535727	SLC27A2	−1.21	4.38E-08
**Metabolic pathways**			
100036592	FUT7	1.27	2.32E-02
100297709	PNPLA3	1.39	1.80E-02
281152	FASN	1.43	3.72E-05
281156	FDPS	1.61	6.71E-07
281174	FUT1	1.31	1.31E-04
281235	IDH1	−1.04	7.66E-11
281274	LDHA	−1.63	9.58E-06
281275	LDHB	−1.37	3.06E-04
281365	ODC1	−1.45	4.60E-11
281767	FDFT1	1.17	1.22E-04
281785	GLUD1	1.01	6.07E-14
282022	PTGS1	1.12	6.45E-03
282645	CHIA	1.08	2.21E-05
282870	CYP1A1	1.27	5.94E-03
286792	UGT1A6	2.17	2.91E-07
327664	PYGM	2.08	7.10E-03
338074	AOX1	1.49	4.94E-05
338471	PC	−1.35	3.70E-19
407159	HMGCR	2.14	7.78E-10
407767	HMGCS1	2.38	4.95E-08
494318	PLA2G2D1	2.20	5.01E-03
497020	ST8SIA5	2.25	2.81E-04
497025	BHMT	−1.77	1.01E-11
503684	HMGCS2	−1.08	2.83E-05
504481	MSMO1	1.69	1.24E-08
505060	CYP51A1	1.83	1.22E-07
505290	MGLL	−1.71	4.70E-23
505315	AMDHD1	−1.90	5.20E-17
505792	MVK	1.21	1.25E-05
505987	GK	−1.68	3.33E-06
506459	ACSS2	1.34	2.73E-03
507323	RIMKLA	−1.46	3.88E-02
507456	AGPAT4	1.32	6.81E-03
508167	RRM2	1.26	1.09E-02
509808	LIPG	−6.63	1.18E-15
509958	MVD	1.30	7.23E-07
510507	CYP7A1	2.40	3.40E-02
511060	EXTL1	1.63	2.61E-07
513221	HMOX1	1.22	3.31E-03
513483	FBP1	−1.28	8.68E-11
513608	ARG1	−1.49	5.04E-06
514293	IDI1	1.16	1.44E-07
514346	SDS	−2.83	1.05E-02
514745	DHCR7	1.55	4.42E-06
515263	ALDOB	−1.04	3.87E-09
515710	HSD17B2	1.14	2.40E-03
515950	HDC	−2.35	7.49E-15
516036	CBR3	−1.17	1.22E-02
516241	CSAD	1.02	7.40E-03
516405	CMBL	1.29	1.80E-23
516539	NANP	−1.06	2.95E-02
517063	AK4	2.64	2.52E-05
518393	AOX4	5.40	1.25E-04
524639	PHOSPHO1	1.77	1.75E-02
526535	SQLE	3.67	1.47E-21
533044	PSAT1	1.09	8.65E-04
533630	PSPH	1.46	3.80E-03
533726	DHCR24	1.54	1.03E-05
534599	OGDH	−1.32	1.85E-05
537451	GYS2	−1.15	2.27E-04
538710	G6PC	−1.10	5.20E-08
539561	GLS2	−1.10	1.69E-03
539606	PYCR1	1.45	4.18E-03
540500	GALNT14	−2.37	7.04E-03
540994	DEGS2	1.40	2.87E-04
613919	GGT6	−1.63	3.13E-02
615205	HAL	−2.12	6.29E-11
615906	LSS	1.36	2.16E-08
616683	BHMT2	−1.02	2.03E-04
616901	CERS6	1.02	8.73E-06
618400	GPT2	−1.02	1.12E-02
785383	ACOT2	−1.50	5.04E-03
**AMPK signaling pathway**			
280924	SCD	2.97	2.68E-10
281152	FASN	1.43	3.72E-05
281239	IGF1	2.04	1.75E-05
281667	CCNA2	1.22	4.90E-02
407159	HMGCR	2.14	7.78E-10
509459	CPT1B	−1.16	2.88E-03
513010	CREB3L3	−1.17	2.11E-09
513483	FBP1	−1.28	8.68E-11
524530	CCND1	1.14	2.27E-03
537451	GYS2	−1.15	2.27E-04
538710	G6PC	−1.10	5.20E-08

### qPCR Analysis of Liver DEGs in Response to Cd

**Figure 8:**
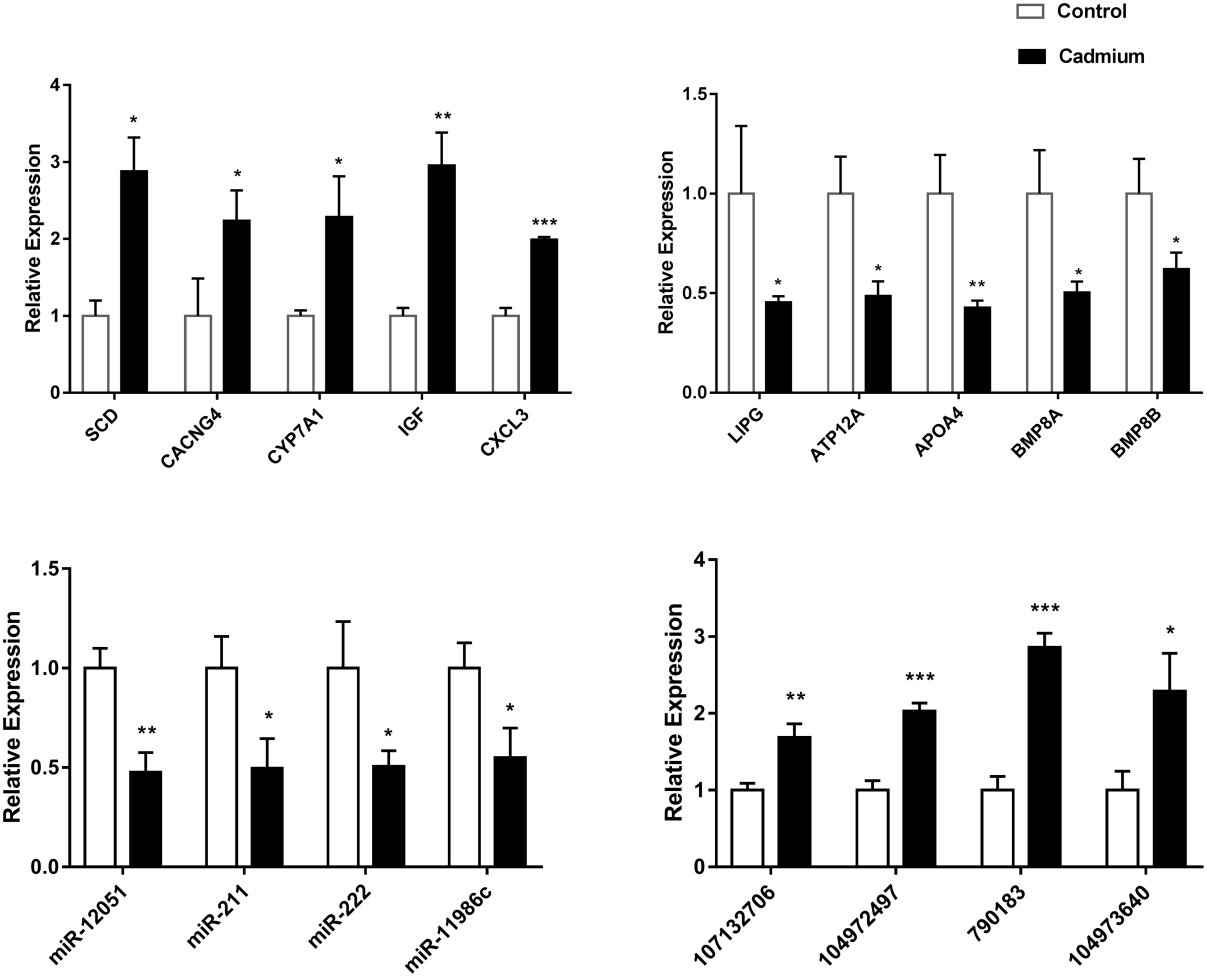
The relative expression of DEGs. The relative expression of the DEGmRNAs (ApoA4, SCD, CYP7A1, CXCL3, LIPG, IGF1, ATP12A, CACNG4, BMP8A, and BMP8B), DEmiRNAs (bta-miR-12051, bta-miR-211, bta-miR-222, and bta-miR-11986c), and DElncRNAs (107132706, 104972497, 790183, and 104973640). Data (*n* = 3) are expressed as the mean ± SEM. For the cadmium-treated group compared with control group: *p < 0.05, **p < 0.01, ***p < 0.001.

Real-time fluorescence quantitative PCR was used to validate the 11 DEmRNAs, four DEmiRNAs, and four DElncRNAs selected for this study. Among the DEmRNAs, stearoyl-CoA desaturase (SCD), voltage-gated channel auxiliary subunit gamma 4 (CACNG4), CYP7A1, insulin-like growth factor 1 (IGF1), and C–X–C motif chemokine ligand 3 (CXCL3) were significantly upregulated. The difference in CXCL3 expression was extremely significant. In addition, LIPG, ATPase H^+^/K^+^ transporting the non-gastric alpha2 subunit (ATP12A), apolipoprotein A (ApoA4), bone morphogenetic protein 8A (BMP8A), and bone morphogenetic protein 8B (BMP8B) were significantly downregulated DEmRNAs. Among the DEmiRNAs, bta-miR-12051, bta-miR-211, bta-miR-222, and bta-miR-11986c were significantly downregulated. Among the DElncRNAs, 107132706, 104972497, 790183, and 104973640 were significantly upregulated. It is worth mentioning that the difference in the expression levels of 104972497 and 790183 was extremely significant (Figure 8). The qRT-PCR results for these DEGs were consistent with the sequencing data.

## Discussion

Hunan is rich in mineral resources, but the problem of Cd-contaminated soil is severe. Cd is a toxic heavy metal with high mobility in soil, and can greatly endanger the health of humans and animals when it enters their bodies through diet and water. The Xiangxi yellow heifer is a special breed in Hunan that feeds on local forage. Therefore, they are highly susceptible to Cd contamination. Chronic Cd exposure is mainly deposited in the liver and can induce liver inflammation (43), and even acute and chronic liver lesions (44). Moreover, redox imbalance, mitochondrial dysfunction, fatty acid oxidation inhibition, and apoptosis are considered the main adverse effects of Cd exposure (26, 45, 46). In this study, the whole transcriptome of liver tissues of Xiangxi yellow heifers fed high and low Cd concentrations was determined, and DEGs were analyzed to study the mechanisms of Cd toxicity in the heifer liver. The critical DEmRNAs involved in Cd detoxification between the control and cadmium-treated groups were analyzed using KEGG and GO analyses.

The predominant toxic effect of Cd exposure is ROS production, which promotes the production of mitochondrial phagosomes that engulf damaged mitochondria (47). Cd exposure has been reported to increase the expression of adenosine 5'-monophosphate (AMP)–activated protein kinase (AMPK) (48). AMPK is also involved in autophagy regulation (49). This is consistent with the KEGG enrichment results of the current study. The effect of Cd on mitochondrial autophagy in the liver of Xiangxi yellow heifers has been demonstrated. SCD is enriched in the AMPK signaling pathway and mediates fatty acid metabolism in the liver and adipogenesis (50). SCD-1, a common isoform of this enzyme (51), is a crucial gene in hepatic steatosis and plays a straightforward role in intracellular fat accumulation in NAFLD. In addition, excessive accumulation of liver fat leads to liver inflammation (52). Research has found that the mRNA levels of SCD-1 are also significantly higher in rats administered with CdCl_2_ in their drinking water (53). NAFLD is associated with elevated mRNA levels of SCD-1. High Cd exposure significantly upregulated SCD expression in our study. This demonstrates that the entry of Cd into the bodies of humans and animals can seriously disrupt the metabolic system and even cause metabolic diseases. In the current study, metabolic pathways were enriched with the most DEmRNAs. The endothelial lipase gene (LIPG) can hydrolyze phosphatidylcholine (PC) in HDL and release free fatty acids (FFAs) (54). In the present study, LIPG expression was significantly downregulated, suggesting that HDL hydrolysis was inhibited in the liver. Thus, cholesterol in the surrounding tissues could not be transported to bile acids in time, and fat accumulated in the liver. Cholesterol 7α-hydroxylase (CYP7A1) is in the metabolic pathway and belongs to the cytochrome P450 enzyme (CYP450) family. It is correlated with inflammatory cytokines and lysosomal function (55, 56). CYP7A1 overexpression strongly induces autophagy (57), and CYP7A1 is required for autophagy induction. In the present study, the expression levels of CYP7A1 mRNA were significantly altered, as shown by qRT-PCR. This indicates that Cd induces autophagy in the liver.

The PPAR signaling pathway was also significantly enriched in the liver. PPARs are a class of nuclear receptors that bind to fatty acids and their derivatives (58). PPAR-α has been reported to have transcriptional regulatory effects on genes involved in gluconeogenesis and lipid transport, and PPAR-γ controls glucose metabolism, adipogenesis, and adipocyte differentiation. Part of PPAR-γ inhibits inflammatory responses by competitively inhibiting inflammatory pathways and inflammatory mediator production (59). PPAR-β promotes hepatic fatty acid oxidation and limits inflammation (60–62). Evidence suggests that PPAR-γ is closely related to the toxicity of heavy metals and that Cr inhibits fibroblast differentiation by reducing PPAR-γ (63). SCD and CYP7A1 genes enriched in the PPAR signaling pathway are closely related to cellular inflammation and adipogenesis in humans and animals. In this study, the mRNA expression levels of SCD and CYP7A1 were significantly upregulated in heifers under chronic Cd exposure. These findings show that this pathway is indeed involved in the toxic effects of heavy metals. In addition to the DEmRNAs in these three significantly different pathways, there were several DEGs related to liver physiological functions after Cd exposure (CACNG4, CXCL3, ATP12A, APOA4, BMP8A, BMP8B, and IGF-1). CACNG4 regulates voltage-gated calcium channels, and studies have shown that Cd exposure increases intracellular Ca^2+^ concentration (64). Elevated Ca^2+^ channel activity leads to increased CACNG4 expression (65, 66). The elevated levels of CACNG4 expression in this study suggest an imbalance in cellular Ca^2+^ concentrations in the liver owing to Cd exposure. CXCL3 is a pro-inflammatory cytokine that is induced alongside MMP12 in mice undergoing oxidative stress (67). CXCL3 exerts its anti-apoptotic effect mainly by counteracting the reversal of the cationic gradient during apoptosis (68). The significant upregulation of CXCL3 in this study demonstrates that Cd causes liver inflammation. ATP12A is a non-gastric, functionally active H^+^/K^+^-ATPase that is expressed mainly in the human and porcine respiratory tract, human prostate tissue, and bone marrow mono-nuclear cells. (69–71). In the present study, ATP12A expression was measured in the livers of heifers. The marked downregulation of ATP12A expression levels suggests that Cd reversed the cationic gradient in the liver, leading to apoptosis. APOA4, a lipid-binding protein, is involved in the synthesis of cholesterol, steroids, and other lipids, and improves glucose homeostasis (72). The anti-inflammatory and antioxidant activities of APOA4 are crucial for maintaining hepatic homeostasis (73, 74). In our study, APOA4 was downregulated, indicating that Cd injury affected fat metabolism, inflammation, and oxidative stress in the liver. Furthermore, BMP8A regulates ROS homeostasis *via* Nrf2 (75), and BMP8B is involved in the regulation of growth traits in heifers (76). In addition, IGF-1 modulates cell proliferation and apoptosis through the PI3/AKT and MAPK pathways (77). Overall, these seven genes were not enriched in significantly different pathways in the transcriptome data. Nevertheless, they indicated that Cd exposure is involved in hepatotoxicity regarding several crucial metabolic pathways and life processes, including inflammation, apoptosis, oxidative stress, and lipid metabolism. These findings provide insight into the mechanisms underlying Cd-induced hepatotoxicity in mammals.

Studies have demonstrated that miRNAs and lncRNAs can regulate the cell cycle, apoptosis, DNA damage, and inflammation in the mechanisms underlying Cd toxicity (78–81). Partial DElncRNAs and DEmiRNAs were selected for further analyses. They were verified by RT-PCR and matched with the data obtained by sequencing, which proved the accuracy and reliability of the sequencing data. The target mRNAs of the DElncRNAs and DEmiRNAs were enriched in the AMPK signaling pathway, glycerophospholipid metabolism, metabolic pathways, and peroxisomes. Therefore, the DElncRNAs, DEmiRNAs, and DEmRNAs identified in this study may be used as biomarkers of Cd toxicity in the future, which is of major research significance.

## Conclusions

In the present study, we determined the gene expression patterns of Cd-exposed livers and DEGs in Xiangxi yellow heifers. The AMPK pathway involved in autophagy regulation, and the PPAR pathway involved in lipid metabolism, were significantly enriched in the livers of Xiangxi yellow cattle fed with high Cd diets. DEmRNAs included the anti-inflammatory and antioxidant APOA4, anti-apoptotic ATP12A, cholesterol metabolism–related LIPG, CXCL3 involved in fatty liver development, cholesterol-metabolizing enzyme CYP7A1, and lipid metabolism–related SCD. DEmiRNAs included bta-miR-12051, bta-miR-211, bta-miR-222, and bta-miR-11986c. DElncRNAs included 107132706, 104972497, 790183, and 104973640. In summary, these genes play a crucial role in Cd-induced liver damage and are critical for future studies of liver diseases associated with Cd exposure.

## Data Availability Statement

The datasets presented in this study can be found in online repositories. The names of the repository/repositories and accession number(s) can be found below: https://www.ncbi.nlm.nih.gov/, PRJNA801770; https://www.ncbi.nlm.nih.gov/, PRJNA801802.

## Ethics Statement

The animal study was reviewed and approved by the Tab of Animal Experimental Ethical Inspection, JLU. Written informed consent was obtained from the owners for the participation of their animals in this study.

## Author Contributions

YW and KY performed the experiments and analyzed the data. All authors read and approved the final version of the manuscript.

## Funding

This work was supported by the Key Research and Development Program of Jilin Province (20210202048NC and 20210202103NC), the Key Research and Development Project of Hunan Province, China (Nos. 2020NK2066 and 2022NK2025), the Construction of Modern Agricultural Industrial Technology System in Hunan Province (Hunan Financial Agriculture Guide 2019 [97]), and the Xiangxi Yellow Cattle Engineering Technology Center (2019TP2010).

## Conflict of Interest

The authors declare that the research was conducted in the absence of any commercial or financial relationships that could be construed as a potential conflict of interest.

## Publisher's Note

All claims expressed in this article are solely those of the authors and do not necessarily represent those of their affiliated organizations, or those of the publisher, the editors and the reviewers. Any product that may be evaluated in this article, or claim that may be made by its manufacturer, is not guaranteed or endorsed by the publisher.
