# Corrigendum: Whole transcriptome profiling of the effects of cadmium on the liver of the Xiangxi yellow heifer

**DOI:** 10.3389/fvets.2023.1135563

**Published:** 2023-01-24

**Authors:** Yameng Wei, Kangle Yi, Caomeihui Shen, Xue Chen, Tariq Iqbal, Maosheng Cao, Tong Chen, Yang Luo, Jianbo Li, Xu Zhou, Chunjin Li, Lu Chen

**Affiliations:** ^1^College of Animal Sciences, Jilin University, Changchun, China; ^2^Grassland and Herbivore Research Laboratory, Hunan Animal Husbandry and Veterinary Research Institute, Changsha, China

**Keywords:** RNA-seq, cadmium, liver, gene ontology, Kyoto Encyclopedia of Genes

In the published article, there was an error with a reference as follows:

21. Eoi: 10.1007/BF02686024dy of the effects of cadmium and copper on fefails to reduce cadmium-induced oxidative damage in rat liver. *Exp Toxicol Pathol*. (2007) 58:367–74. doi: 10.1016/j.etp.2006.11.006.

The correct reference is below:

21. Eşrefoglu M, Gül M, Dogru MI, Dogru A, Yürekli M. Adrenomedullin fails to reduce cadmium-induced oxidative damage in rat liver. *Exp Toxicol Pathol*. (2007) 58:367–74. doi: 10.1016/j.etp.2006.11.006.

The authors apologize for this error and state that this does not change the scientific conclusions of the article in any way. The original article has been updated.

