# Supplementary material for: Whole Transcriptome Profiling of the Effects of Cadmium on the Liver of the Xiangxi Yellow Heifer
Source: Front Vet Sci. 2022 Apr 14;9:846662. doi: 10.3389/fvets.2022.846662 (PMC9047687; doi:10.3389/fvets.2022.846662)
Supplement: Supplementary file 1 [file Table_1.docx]

Supplementary Material

# Table S1

Primer sequences for qPCR used in this study

| Gene Name | Primer (5’-3’) | |
| --- | --- | --- |
| actin | F | CCCTGAGCGCAAGTACTCCG |
|  | R | GACTCATCGTACTCCTGCTTGCT |
| miR-12051 | F | CTCAACTGGTGTCGTGGA |
|  | R | GCCGAGACTCCTGCAGCATCT |
|  | RT | CTCAACTGGTGTCGTGGATCGGAAC |
| miR-211 | R | GCCGAGTTCCCTTTGTCATC |
|  | RT | CTCAACTGGTGTCGTGGAGGCAAAG |
| miR-222 | R | GCCGAGAGCTACATCTGGCTA |
|  | RT | CTCAACTGGTGTCGTGGAACCCAG |
| miR-11986c | R | GCCGAGGAACTGAGTCCTTTG |
|  | RT | CTCAACTGGTGTCGTGGATCTTTTC |
| 107132706 | F | ATTAGAGTATCAGCCTTGTCA |
|  | R | CTGAGCTACTAAGCACCCA |
| 104972497 | F | GAATCCATCAATTCCGCTT |
|  | R | TACTTGGGCTCTAGGCTAC |
| 790183 | F | CAGGACCTTCCGGTGCCATG |
|  | R | AACTGTCCCTCTAAGACTGCC |
| 104973640 | F | ATCCTCGATCTGAACTGGTA |
|  | R | TGGAGGCCCTAGTTACTCA |
| SCD | F | TCCGACCTAAGAGCCGAGA |
|  | R | CGTTTCATCCCACAGATACCAT |
| CACNG4 | F | GTCTTCCCCATCCTGAGCACCA |
|  | R | CACGATGTTGTTCTTGCGGCTGT |
| CYP7A1 | F | CAAGTCACTCCTCGCAGTC |
|  | R | TTGACCAGCATTCTCTAGTGT |
| IGF1 | F | CCATCACATCCTCCTCGCATC |
|  | R | TGAAATAAAAGCCCCTGTCTCCG |
| CXCL3 | F | ACATACAGAGCGTGAAGGTGAC |
|  | R | AGGGTTGAGACAAGCTTCCT |
| LIPG | F | GGCTCACCAGCTTTACGTAG |
|  | R | TAGCCAATCAAGTGAACATTCCC |
| ATP12A | F | TCATGTCCAGTTTCCGCAAG |
|  | R | CCTTTAATCTCCACTATGTCCCC |
| APOA4 | F | GGGAAGTGAGCACCTACACGGAC |
|  | R | CCAGCTCCTTCCGAATCTCCT |
| BMP8A | F | GGACTCCTGCATGAACGCCACCA |
|  | R | CAGCACGCCTTGGGGACCG |
| BMP8B | F | CTCCTTCCCGCTGGACTCCT |
|  | R | GTAGAGCACGGAAGTGGCGCTCA |
